# Supplementary material for: Modeling glioblastoma heterogeneity as a dynamic network of cell states
Source: Mol Syst Biol. 2021 Sep 16;17(9):e10105. doi: 10.15252/msb.202010105 (PMC8444284; doi:10.15252/msb.202010105)
Supplement: Supplementary file 5 — Source Data for Figure 3 [file MSB-17-e10105-s001.zip › Figure3A_sourcedata/GSEA_3065/hallmarks_state6.GseaPreranked.1623416308702/HALLMARK_WNT_BETA_CATENIN_SIGNALING.html]

Details for gene set HALLMARK\_WNT\_BETA\_CATENIN\_SIGNALING[GSEA]

|  || Dataset | state6 |
| Phenotype | NoPhenotypeAvailable |
| Upregulated in class | na\_pos |
| GeneSet | HALLMARK\_WNT\_BETA\_CATENIN\_SIGNALING |
| Enrichment Score (ES) | 0.38591453 |
| Normalized Enrichment Score (NES) | 1.2565553 |
| Nominal p-value | 0.1505618 |
| FDR q-value | 0.22294812 |
| FWER p-Value | 0.857 |
Table: GSEA Results Summary

  

Fig 1: Enrichment plot: HALLMARK\_WNT\_BETA\_CATENIN\_SIGNALING      
 Profile of the Running ES Score & Positions of GeneSet Members on the Rank Ordered List

  

| PROBE | GENE SYMBOL | GENE\_TITLE | RANK IN GENE LIST | RANK METRIC SCORE | RUNNING ES | CORE ENRICHMENT || 1 | CCND2 |  |  | 32 | 0.269 | 0.1695 | Yes |
| 2 | TP53 |  |  | 116 | 0.205 | 0.2922 | Yes |
| 3 | CSNK1E |  |  | 580 | 0.116 | 0.3140 | Yes |
| 4 | HDAC5 |  |  | 595 | 0.114 | 0.3859 | Yes |
| 5 | CUL1 |  |  | 1195 | 0.075 | 0.3663 | No |
| 6 | CTNNB1 |  |  | 1461 | 0.066 | 0.3783 | No |
| 7 | MYC |  |  | 2081 | 0.045 | 0.3370 | No |
| 8 | JAG1 |  |  | 2234 | 0.040 | 0.3456 | No |
| 9 | NCSTN |  |  | 2327 | 0.038 | 0.3596 | No |
| 10 | NCOR2 |  |  | 2497 | 0.033 | 0.3618 | No |
| 11 | RBPJ |  |  | 3008 | 0.022 | 0.3178 | No |
| 12 | ADAM17 |  |  | 3218 | 0.017 | 0.3051 | No |
| 13 | GNAI1 |  |  | 3240 | 0.017 | 0.3135 | No |
| 14 | NOTCH1 |  |  | 3459 | 0.012 | 0.2962 | No |
| 15 | AXIN1 |  |  | 3516 | 0.011 | 0.2969 | No |
| 16 | HEY1 |  |  | 3541 | 0.011 | 0.3009 | No |
| 17 | DVL2 |  |  | 3925 | 0.002 | 0.2584 | No |
| 18 | NUMB |  |  | 4091 | -0.001 | 0.2403 | No |
| 19 | MAML1 |  |  | 4142 | -0.002 | 0.2360 | No |
| 20 | KAT2A |  |  | 4615 | -0.011 | 0.1892 | No |
| 21 | SKP2 |  |  | 4907 | -0.017 | 0.1668 | No |
| 22 | TCF7 |  |  | 5094 | -0.020 | 0.1586 | No |
| 23 | LEF1 |  |  | 5275 | -0.024 | 0.1534 | No |
| 24 | PTCH1 |  |  | 5310 | -0.025 | 0.1653 | No |
| 25 | HDAC2 |  |  | 5329 | -0.025 | 0.1793 | No |
| 26 | PSEN2 |  |  | 6403 | -0.047 | 0.0874 | No |
| 27 | DKK1 |  |  | 8749 | -0.288 | 0.0054 | No |
Table: GSEA details [plain text format]

  

Fig 2: HALLMARK\_WNT\_BETA\_CATENIN\_SIGNALING: Random ES distribution      
 Gene set null distribution of ES for **HALLMARK\_WNT\_BETA\_CATENIN\_SIGNALING**
